# Early stage transplantation of bone marrow cells markedly ameliorates copper metabolism and restores liver function in a mouse model of Wilson disease

**DOI:** 10.1186/1471-230X-11-75

**Published:** 2011-06-15

**Authors:** Xi Chen, Shihui Xing, Yanqing Feng, Songlin Chen, Zhong Pei, Chuhuai Wang, Xiuling Liang

**Affiliations:** 1 Department of Rehabilitation Medicine, The First Affiliated Hospital, Sun Yat-Sen University, No. 58 Zhongshan Road 2, Guangzhou 510080, PR China; 2 Department of Neurology, The First Affiliated Hospital, Sun Yat-Sen University, No. 58 Zhongshan Road 2, Guangzhou 510080, PR China

## Abstract

**Background:**

Recent studies have demonstrated that normal bone marrow (BM) cells transplantation can correct liver injury in a mouse model of Wilson disease (WD). However, it still remains unknown when BM cells transplantation should be administered. The aim of this study was to investigate the potential impact of normal BM cells transplantation at different stages of WD to correct liver injury in toxic milk (tx) mice.

**Methods:**

Recipient tx mice were sublethally irradiated (5 Gy) prior to transplantation. The congenic wild-type (DL) BM cells labeled with CM-DiI were transplanted via caudal vein injection into tx mice at the early (2 months of age) or late stage (5 months of age) of WD. The same volume of saline or tx BM cells were injected as controls. The DL donor cell population, copper concentration, serum ceruloplasmin oxidase activity and aspartate aminotransferase (AST) levels in the various groups were evaluated at 1, 4, 8 and 12 weeks post-transplant, respectively.

**Results:**

The DL BM cells population was observed from 1 to 12 weeks and peaked by the 4^th ^week in the recipient liver after transplantation. DL BM cells transplantation during the early stage significantly corrected copper accumulation, AST across the observed time points and serum ceruloplasmin oxidase activity through 8 to 12 weeks in tx mice compared with those treated with saline or tx BM cells (all *P *< 0.05). In contrast, BM cells transplantation during the late stage only corrected AST levels from 4 to 12 weeks post-transplant and copper accumulation at 12 weeks post-transplant (all *P *< 0.05). No significant difference was found between the saline and tx BM cells transplantation groups across the observed time points (*P *> 0.05).

**Conclusions:**

Early stage transplantation of normal BM cells is better than late stage transplantation in correcting liver function and copper metabolism in a mouse model of WD.

## Background

Wilson disease (WD) is an autosomal recessive disease that is caused by a loss-of-function mutation in the ATP7B gene and is characterized by hypoceruloplasminemia and excessive accumulation of copper in various organs [1]. The accumulation of copper in turn leads to serious chronic liver injury and neurological dysfunction [2]. Copper chelating agents are widely used to restore hepatic copper homeostasis, but they must be administrated over a lifetime and have little effect in severe cases. Orthotopic liver transplantation allows the recipient to metabolize copper correctly, preventing the progression of disease, and it is especially suited for patients with liver failure [3,4]. Unfortunately, orthotopic liver transplantation is mostly unavailable because of several limitations such as a lack of donors, rejection and high cost [5,6].

Recent evidence has indicated that hepatocyte transplantation not only provides temporary liver function but also cures certain metabolic conditions in the rat model of WD [7,8]. Consistently, our previous study has demonstrated that embryonic hepatocytes are capable of differentiating into mature hepatocytes in vivo and partially correct abnormalities of copper metabolism after intraspleenic transplantation of homogeneous embryonic hepatocytes in toxic milk (tx) mice [9]. However, hepatocytes that are used for transplantation have to be obtained from the limited supply of donors. Thus, it would be highly desirable to have a readily available alternate source of cells.

Hepatocytes can be replaced by bone marrow (BM) cells under suitable circumstances in animals and humans [10]. Several recent studies have demonstrated that BM cells contribute to the renewal of hepatocytes and have the potential to treat liver injury, including acute or chronic liver failure [11,12]. BM cells transplantation can partially reduce liver copper levels and correct liver disease in tx mice at five months post-transplant, and the beneficial effects of BM cells transplantation are similar to those obtained from normal congenic liver cells [13]. More recently, BM cells transplanted into tx mice have been shown to engraft in the liver and produce partial metabolic disease correction via reducing liver copper and increasing ceruloplasmin oxidase activity, although this effect may not be sustained over a 9-month period post-transplant [14]. However, it still remains unclear when BM cells transplantation should be administrated to correct liver dysfunction in mice with WD.

The tx mouse is a naturally occurring genetic and phenotypic model of WD derived from the congenic wild-type (DL) mouse [15]. The tx mouse has an equivalent point mutation in the ATP7B gene to humans, which causes early copper accumulation in the liver and late accumulation in other tissues [15,16]. Previously, we have confirmed that tx mice present the early stage characteristics of WD at 2 months of age and arrive the peak stage of WD at 4 to 5 months of age in terms of copper metabolism and liver function [17]. In the present study, we aimed to further investigate whether BM cells transplantation at different stages of WD has potential implications in copper metabolism and correction of liver function in tx mice.

## Methods

### Mouse Strains and Animal Husbandry

The experimental protocol was approved by the local ethical committee for animal research, and all procedures involving the animals were conducted according to institutional guidelines. DL and homologous tx mice were kindly donated by Dr. Julian Mercer (Deakin University, Australia) and used as BM cells donors and recipients, respectively. All animals used in the study were bred and maintained in the mouse facility at Sun Yat-Sen University under 14-h light and 10-h dark cycles. Drinking water and normal diet were regularly maintained. Tx mice were allocated to one of the six groups as follows: 2-month-old mice treated with DL BM cells transplantation, 5-month-old mice treated with DL BM cells transplantation, 2- or 5-month-old mice treated with saline as a blank control and 2- or 5-month-old mice treated with tx BM cells as a transplantation control (40 mice per group, female:male = 3:2).

### BM Cells Extraction and Transplantation

Four- to five-week-old congenic male DL mice were selected for BM cells extraction. The femur and tibia were flushed with D-hanks media (containing 8.00 g NaCl, 0.40 g KCl, 0.12 g Na_2_HPO_4_·12H_2_O, 0.06 g KH_2_PO_4 _and 1.00 g anhydrous dextrose in 1 L distilled water) to extract BM cells. The cell suspension was filtered through nylon mesh and centrifuged at 900 × g for 10 min at room temperature (RT). Red blood cells were lysed by the addition of blood cell lysis buffer (Solarbio Company, China); a buffer volume corresponding to 4 times the volume of cells was used. Tubes were placed on ice for 15 min and then centrifuged at 450 × g for 10 min at 4°C, followed by resuspension in D-hanks solution. To trace the donor BM cells, CM-DiI (Molecular Probes Company, USA) was used to label the BM cells. In brief, BM cells were incubated with CM-DiI (1:250) for 5 min at RT, followed by an additional 15 min at 4°C and then centrifuged at 1500 × g for 5 min at RT. The cells were washed with D-hanks solution (2 x) and centrifuged at 1500 × g for 5 min at RT. Thereafter, the cells were resuspened in D-hanks solution to a final concentration of 6 × 10^7^/ml. Before transplantation, the number and viability of the cells were estimated using the trypan blue exclusion test, which was used as the standard protocol. Viable cells accounted for 98% of the total cells. Tx BM cells were also prepared as described above.

All recipient tx mice were sublethally irradiated (5 Gy) three days before transplantation. To transplant cells, animals were anesthetized with 10% chloral hydrate (3 ml/kg body weight). Resuspended DL BM cells at a dose of 0.2 ml were intravenously injected into 2- or 5-month-old tx mice. In saline or tx BM cells control groups, 2- or 5-month-old tx mice received the same volume of saline or tx BM cells instead of DL BM cells according to the same procedures described above.

### Tissue preparation

Animals were intracardiacally perfused with 0.9% saline prior to sample collection to ensure the removal of transplanted cells in the blood. Female recipient mice from each group were sacrificed at 1, 4, 8 and 12 weeks after transplantation for DNA extraction (n = 6 for each time point). Genomic DNA was extracted according to the protocol provided with the DNA extraction kit with modification. Briefly, under deep anesthesia with 10% chloral hydrate (5 ml/kg body weight), fresh liver tissue was extracted from the female recipient mice and was minced and placed in lysis buffer (50 mmol/l Tris, pH 7.5, 100 mmol/l EDTA, 100 mmol/l NaCl, 1% sodium dodecyl sulfate containing proteinase K (0.5 mg/ml) and incubated at 55°C overnight. The lysate was allowed to stand at RT for 30 min to equilibrate to RT and was then centrifuged at 4500 × g for 10 min. The supernatant was then placed into a filtration column and centrifuged at 9000 × g for 5 min. After washing to remove contaminants, DNA was bound to the spin column membrane by centrifugation at 9000 × g for 10 min. The spin column was allowed to air dry for 5 min. DNA was eluted with 200 μl TE buffer (pH 7.5) by centrifugation into a collection tube at 9000 × g for 5 min. The purified DNA was prepared for PCR. Ten recipient mice from each group were sacrificed at each time point for the analysis. Blood samples were collected in heparin-containing tubes and centrifuged at 3500 × g for 5 min to obtain serum for ceruloplasmin oxidase activity and aspartate aminotransferase (AST) assessment. The liver sections from the recipient mice (10-μm thick) were sectioned on a cryostat (CM1900; Leica, Heidelberger, Nussloch, Germany) for histological evaluation or immunohistochemistry. Additional tissues from liver, brain and kidney were all stored for trace copper analysis.

### Polymerase Chain Reaction for Sry Gene

Donor cell repopulation was also analyzed using polymerase chain reaction (PCR) amplification of the sex-determining Sry genes region in male to female transplants. The primer sequences for the Sry genes were 5'-TGGGACTGGTGACAATTGTC-3' (forward) and 5'-GAGTACAGGTGTGCACCTCT-3' (reverse), with a predicted product of 444 bp. The β-actin gene was used as a housekeeping positive control. The primer sequences for the β-actin genes were 5'-ATGGATGACGATATCGCT-3' (forward) and 5'-ATGAGGTAGTCTGTCAGG-3' (reverse), with a predicted product of 1110 bp. PCR was performed for 30 cycles with denaturing at 94°C for 1 min, annealing at 55°C for 1 min and extension at 72°C for 1 min. Engraft and cell repopulation were expressed as ratios of the Sry genes level to the corresponding β-actin genes level. The ratios of SRY to β-actin bands were analyzed with Image pro plus imaging analysis software.

### Histopathology and Immunohistochemistry

Sections were stained with hematoxylin and eosin (HE) for histopathological examination using standard methods and were stained with Masson's trichrome (fibrosis-specific staining) to evaluate the degree of liver fibrosis as previously described [18]. The histological features were independently assessed by 2 pathologists who were blinded to the other details of the experiment. The CM-DiI fluorescence intensity was monitored in the liver sections to determine the existence of donor cells in the recipient liver. To confirm the contribution of BM cells to the renewal of hepatocytes, CK-18/CM-DiI double fluorescence intensity was assessed. For immunostaining of CK-18, sections were blocked with 3% normal horse serum and 0.1% Triton X-100 in 0.01M PBS for 1 h at RT. Then, the sections were incubated with mouse anti-CK18 (1:400, Chemicon International, Temecula, CA, USA) as the primary antibody overnight at 4°C. After rinsing, FITC-conjugated goat anti-mouse IgG (1:400, Jackson Immunoresearch Laboratories, USA) was then applied for 1 h at RT. Fluorescence signals were detected using a microscope (BX51; Olympus, Tokyo, Japan) at excitation/emission wavelengths of 492/510 nm (FITC, green) and 550/570 nm (CM-DiI, red).

### Copper Measurement

The stored liver, brain, and kidney tissues at each time point were baked to a permanent weight and analyzed based on atomic absorption spectrophotometry according to the method previously described [19]. Results were expressed in mg/kg dry weight of tissue.

### Serum Ceruloplasmin Oxidase Activity and AST Measurement

The ceruloplasmin oxidase activity in serum using o-dianisidine dihydrochloride was evaluated as previously reported [20]. The AST levels in serum were measured using a 7170A biochemical analyzer according to standard protocols.

### Statistical Analysis

Data were presented as mean ± SD. The normal distribution was tested with Shapiro-Wilk test, and data were statistically analyzed using ANOVA. Probability values of <0.05 were considered to be statistically significant.

## Results

### Engraftment and repopulation after transplantation

**Figure 1:**
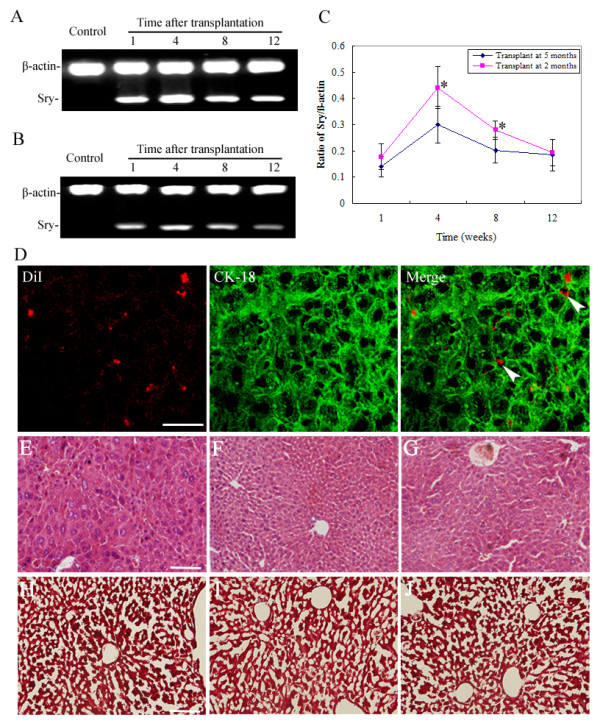
**Engraftment and population after bone marrow (BM) cells transplantation**. The DL donor transplant cells population and expansion were evaluated using PCR for the Sry genes in female tx mice livers in different groups. (A and B) The presence of male DL Sry fragments was confirmed in tx mice treated with DL BM cells transplantation at 2 and 5 months of age respectively. Lane 1: saline-treated mice; lane 2-5: tx mice at 1, 4, 8 and 12 weeks post-DL BM cells transplantation. (C) Ratios of the Sry genes to beta-actin levels were calculated in mice treated with DL BM cells transplantation at 2 and 5 months of age at different time points (*n *= 6). Values were mean ± SD. Data showed a normal distribution and were analyzed using ANOVA. *P < 0.05, compared with animals from the 5 months of age group. (D) Representative immunofluorescent staining for CK-18 in the recipient liver showed that CM-DiI labeled donor BM cells (red) were stained positive for the hepatocellular antigen CK-18 (green) at 4 weeks post-transplant (merged fluorescence is indicated by the arrow). Scale bar: 50 μm. (E-G) HE staining showed the hepatic structures in saline or tx BM cells or the DL BM cells treated groups. (H-J) Masson's trichrome staining showed no obvious liver fibrosis in saline or tx BM cells or the DL BM cells treated groups. Scale bar: 100 μm.

Engraftment and repopulation of BM cells were defined by the presence of donor DNA in female recipient tissues, as characterized by the amplification of the Sry genes. In the saline-control mice, the liver Sry genes were undetectable at all the observed time points. Liver engraftment with DL BM cells was found in female mice treated with male DL BM cells at 2 and 5 months of age (Figure 1 A and 1B). The expression of the Sry gene increased during the first week, peaked at 4 weeks and then decreased across the observed time points after transplantation. Furthermore, the BM cells population in the early stage transplant group was much higher than those in the late stage transplant group at 4 and 8 weeks after transplant (for both, *P *< 0.05; Figure 1C). CK-18 was used as the specific marker for mature hepatocytes. CM-DiI/CK-18 positive cells were observed in both the sinus and parenchyma of the recipient liver as early as the first week and lasted 12 weeks following transplantation in tx mice (Figure 1D). A low CM-DiI fluorescence intensity was detected in kidney and brain (data not shown). Additionally, HE staining showed destroyed hepatic tissues and hepatocyte swelling with vacuoles in the nucleus in the saline and tx BM cells control groups and the DL BM cells treated groups (Figure 1E-G). However, liver fibrosis that was evaluated with Masson's trichrome staining shows no obvious changes across the three groups (Figure 1H-J).

### Effect of BM cells transplantation on the liver copper concentration

**Figure 2:**
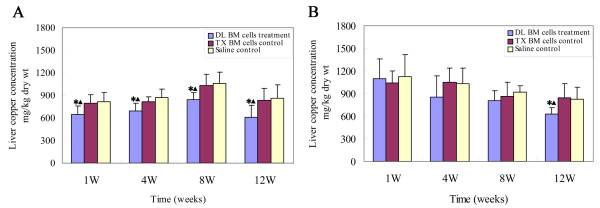
**Effect of BM cells transplantation on copper accumulation in the liver**. The liver copper levels were measured by atomic absorption spectrophotometry in recipient livers from tx mice at 1, 4, 8 and 12 weeks post-transplant. (A) Copper levels in mice treated with DL BM cells or saline or tx BM cells transplantation at 2 months of age. (B) Copper levels in mice treated with DL BM cells or saline or tx BM cells transplantation at 5 months of age (*n *= 10). Values were mean ± SD and analyzed using ANOVA. *P < 0.05, compared with the saline-treated group; ▲*P *< 0.05, compared with the tx BM cells treated group.

The liver copper concentration decreased by approximately 21.2 ± 8.2% at the 1^st ^week, 20.3 ± 5.9% by the 4^th ^week, 19.7 ± 7.4% by the 8^th ^week and 29.7 ± 8.3% by the 12^th ^week post-transplant in mice treated with DL BM cells at 2 months of age compared to those in age-matched mice with saline transplantation, respectively (for all, *P *< 0.05). Consistently, DL BM cells transplantation significantly reduced the liver copper concentration at the corresponding time points post-transplant compared to the tx BM cells control group (for all, *P *< 0.05). However, despite the presence of donor cells in mice treated with DL BM cells at 5 months of age over the observed period, the liver copper levels decreased approximately 24.2% and 25.9% at the 12^th ^week after DL BM cells transplantation as compared to the saline and tx BM cells control groups, respectively (for both, *P *< 0.05; Figure 2A). The liver copper concentration in mice treated with DL BM cells transplantation at 5 months of age was not significantly different from that in mice treated with saline or tx BM cells at 1, 4 and 8 weeks after transplantation (for all, *P *> 0.05; Figure 2B). Notably, there was some reduction in the copper concentration in brain tissue during the 4^th ^week in mice treated with DL BM cells transplantation at 2 months of age (*P *< 0.05); however, this effect was not evident in mice treated with DL BM cells transplantation at 5 months of age. No significant differences in the kidney copper concentrations were found between the DL BM cells transplant group and the control groups (*P *> 0.05, data not shown). Additionally, there was no significant difference between the saline and tx BM cells transplantation groups across the observed time points (*P *> 0.05).

### Effect of BM cells transplantation on the serum ceruloplasmin oxidase activity

**Figure 3:**
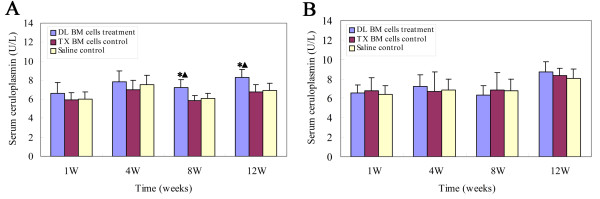
**Effect of BM cells transplantation on the ceruloplasmin oxidase activity**. The serum ceruloplasmin oxidase activity was measured using an o-dianisidine dihydrochloride test in tx mice at 1, 4, 8 and 12 weeks post-transplant. (A) Ceruloplasmin oxidase activity in mice treated with DL BM cells or saline or tx BM cells transplantation at 2 months of age. (B) Ceruloplasmin oxidase activity in mice treated with DL BM cells or saline or tx BM cells transplantation at 5 months of age (*n *= 10). Values were mean ± SD and analyzed using ANOVA. *P < 0.05, compared with the saline-treated group; ▲*P *< 0.05, compared with the tx BM cells treated group.

The serum ceruloplasmin oxidase activity in mice receiving transplantation of DL BM cells at 2 months of age was significantly higher than that of those treated with saline or tx BM cells from 8 to 12 weeks post-transplant, respectively (*P *< 0.05, Figure 3A). However, DL BM cells transplantation did not significantly increase the serum ceruloplasmin oxidase activity in mice receiving transplantation at 5 months of age throughout all the observed time points as compared to those treated with saline or tx BM cells (*P *> 0.05, Figure 3B). No significant difference was found between the saline and tx BM cells transplantation groups across the observed time points (*P *> 0.05).

### Effect of BM cells transplantation on the AST levels

**Figure 4:**
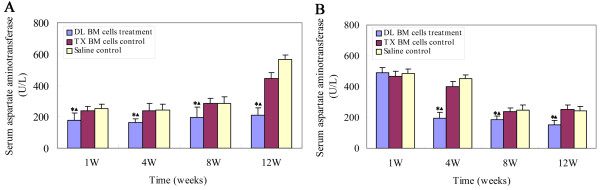
**Effect of BM cells transplantation on the AST levels**. The serum AST levels were measured by a 7170A biochemical analyzer to represent an index of liver function in tx mice at 1, 4, 8 and 12 weeks post-transplant. (A) Serum AST levels in mice treated with DL BM cells or saline or tx BM cells transplantation at 2 months of age. (B) Serum AST levels in mice treated with DL BM cells or saline or tx BM cells transplantation at 5 months of age (*n *= 10). Values were mean ± SD and analyzed using ANOVA. *P < 0.05, compared with the saline-treated group; ▲*P *< 0.05, compared with the tx BM cells treated group.

The serum AST level was measured in different groups to confirm the recovery of liver function. In the 2-month-old mice transplant group, DL BM cells significantly reduced the serum AST levels during the 1^st ^week (approximately 30.4%), and the reduction persisted for at least 12 weeks (63.0%) post-transplant as compared to the saline control group at the corresponding time points (*P *< 0.05, Figure 4A). Compared to the tx BM control group, a significant reduction in the AST levels was also found at 1 to 12 weeks post-transplant (*P *< 0.05, Figure 4A). However, a significant reduction in the AST level in mice receiving transplantation of DL BM cells at 5 months of age was only found at 4 to 12 weeks post-transplant as compared to the saline and tx BM cells control groups, despite the presence of donor cells across the observed time points (*P *< 0.05, Figure 4B). No significant difference was found between the saline and tx BM cells transplantation groups at different time points (*P *> 0.05).

## Discussion

In the present study, we have investigated the potential effects of normal BM cells transplantation at different stages of WD on the correction of liver function and copper metabolism in tx mice. We found that DL BM cells transplantation at 2 months of age corrects the copper concentration and the AST levels in tx mice at 1 to 12 weeks post-transplant; however, these effects were not significant in tx mice receiving DL BM cells transplantation at 5 months of age. The results indicate that early stage transplantation of BM cells has a greater potential for the correction of liver function and copper metabolism in mice with WD.

Recently, several studies have reported that BM cells transplantation can partially correct liver disease in a WD model [13,14]. Only long-term (5 or 9 months) effects of BM cells transplantation in correcting liver injury have been highlighted in these studies. However, the potential effects of BM transplantation during different stages of the disease on correcting liver function remain undetermined. We found that the liver copper concentration and liver function was significantly corrected at 1 to 12 weeks following DL BM cells transplantation, indicating that normal BM cells transplantation may ameliorate liver damage within a short period following treatment. Importantly, early transplantation of BM cells (at 2 months of age) was more effective for the donor cell population and liver function correction in tx mice. The underlying mechanisms may be related to the variable selective pressure of liver damage. Selective pressure in the form of liver injury has been proven to be required for donor cell engraftment of the liver [10]. However, the toxic effects of excessive copper accumulation or an unsuitable hostile microenvironment from severe liver damage have been conceived to impede transplanted cells engraftment and the proliferation of donor cells, as indicated in the Long-Evans Cinnamon rat model [21-23]. Consistent with previous studies [24], we have revealed that the accumulation of copper under physiological conditions of tx mice peaked by 4 months of age, which is equal to 8 weeks post-transplant in the 2-month transplant group. Then the liver copper concentration of tx mice decreased gradually to half of the peak concentration from 15 to 19 months of age [17]. The control group consistently showed a reduction in the hepatic copper concentration at 12 weeks post-transplant in the present study. Thus, the liver microenvironment of 2-month-old tx mice was more fit for the donor cells planting and proliferation compared to that of the 5-month-old tx mice.

Repopulation of normal BM cells in recipient livers has been well documented in models of WD [13,14]. Based on the previous literature [25], Sry genes in Y-chromosomes were used to verify the repopulation of donor cells in the present study. We found that a marked proliferation of donor BM cells occurred as early as at the 1^st ^week and peaked by the 4^th ^week post-transplant. Previously, donor BM cells have been reported to significantly repopulate at the 20^th ^week post-transplant with correction of liver damage in tx mice. The major discrepancies mainly lie in the different study designs. BM cells transplantations were performed at 3 to 4 months, and the populations of donor cells and liver injury rescues were generally evaluated at 5 and 9 months post-transplant in the previous studies [13,14].

Accumulating evidence indicates that BM cells have great differentiation plasticity and can differentiate into many different types of tissue cells [26]. Recently, transplanted BM cells have been shown to repopulate as hepatic cells under certain circumstances in liver disease models [27,28]. To evaluate donor cells in the liver, CM-DiI was employed as a long-term tracing marker, in that CM-DiI has the advantages of being a photo-stable fluorescence dye with excellent cellular retention and minimal cytotoxicity [29-31]. We found that CM-DiI positive donor cells appear in liver sinuses as early as the 1^st ^week post-transplant and extended into liver parenchyma thereafter. Furthermore, some donor cells expressed hepatocyte-related CK18 post-transplant, suggesting the formation of hepatocyte-like cells. In addition, there has been increasing evidence that transplanted marrow cells may regenerate the liver by cell fusion [32,33]. Donor hematopoietic cells can fuse with host hepatocytes and express both donor and host genes, which is consistent with polyploid genome formation by the fusion of host and donor cells [33]. Interestingly, CM-DiI fluorescence was present within some hepatocytes in the current study, which suggests the possibility of cell fusion. Therefore, transplanted BM cells may engraft in the recipient liver and function to improve liver injury by differentiation or cell fusion mechanisms. However, the underlying mechanisms were not investigated in the present study. Further studies are needed to elucidate the cell type involved in partial disease correction and cell fusion in mice modeling WD.

Copper accumulation is common in basal ganglion and contributes to the neurological impairment in patients with WD. Consistent with previous studies [13,14], BM cells transplantation, to some degree, reduced the copper accumulation in brain tissue. However, whether lowering copper levels can correct neurological symptoms was not evaluated in this study because neurological symptoms cannot be tested in tx mice [16]. Further studies are needed to determine the potential association between the reduction in copper accumulation and the neuron injury following BM cells transplantation.

## Conclusions

In summary, the present study suggests that normal BM cells transplantation at an early stage of WD may be critical for accelerating correction of liver injury in mice with WD.

## Competing interests

The authors declare that they have no competing interests.

## Authors' contributions

CX performed the BM cells extraction, transplantation and immunochemistry assessments and participated in the sequence alignment. XSH performed the statistical analysis and drafted the manuscript. FYQ performed the tissue preparation and performed PCR for the Sry genes. CSL participated in the copper, serum ceruloplasmin oxidase activity and AST measurement. PZ participated in the sequence alignment and revised the manuscript. WCH participated in the design of the study and the sequence alignment. LXL conceived the study and participated in its design. All authors read and approved the final manuscript.

## Pre-publication history

The pre-publication history for this paper can be accessed here:

http://www.biomedcentral.com/1471-230X/11/75/prepub
